# Superior Neutralizing Antibody Response and Protection in Mice Vaccinated with Heterologous DNA Prime and Virus Like Particle Boost against HPAI H5N1 Virus

**DOI:** 10.1371/journal.pone.0016563

**Published:** 2011-01-28

**Authors:** Heng Ding, Cheguo Tsai, Ramona Alikiiteaga Gutiérrez, Fan Zhou, Philippe Buchy, Vincent Deubel, Paul Zhou

**Affiliations:** 1 Unit of Anti-Viral Immunity and Genetic Therapy, Key Laboratory of Molecular Virology and Immunology, Institut Pasteur of Shanghai, Chinese Academy of Sciences, Shanghai, China; 2 Institut Pasteur in Cambodia, Phnom Penh, Cambodia; WIV-Pasteur Institute, Belgium

## Abstract

**Background:**

Although DNA plasmid and virus-like particle (VLP) vaccines have been individually tested against highly pathogenic avian influenza (HPAI) H5N1 viruses, the combination of both vaccines into a heterologous prime-boost strategy against HPAI H5N1 viruses has not been reported before.

**Methodology/Principal Findings:**

We constructed DNA plasmid encoding H5HA (A/Shenzhen/406H/06, subclade 2.3.4) and generated VLP expressing the same H5HA and N1NA. We then compared neutralizing antibody responses and immune protection elicited with heterologous DNA-VLP, homologous DNA-DNA and VLP-VLP prime-boost strategies against HPAI H5N1 viruses in mice. We demonstrate that DNA-VLP elicits the highest neutralizing antibody titers among the three prime-boost strategies, whereas DNA-DNA elicits higher neutralizing antibody titers than VLP-VLP. We show that although all three prime-boost strategies protect mice from death caused by 10 MLD_50_ of homologous and heterologous H5N1 challenge, only DNA-VLP and DNA-DNA protect mice from infection as manifested by no weight loss and no lung pathology. In addition, we show that although DNA-VLP and DNA-DNA protect mice from death caused by 1,000 MLD_50_ of homologous H5N1 challenge, only DNA-VLP protects mice from infection. Moreover, we show that after 1,000 MLD_50_ of heterologous H5N1 challenge, while all mice in PBS, VLP-VLP and DNA-DNA died, 3 of 6 mice in DNA-VLP actually survived. Finally, we show that DNA-VLP completely protects mice from infection after 1,000 MLD_50_ of homologous H5N1 challenge even when the challenge was administrated at 60 days post the boost.

**Conclusions/Significance:**

These results provide strong support for clinical evaluation of heterologous DNA-VLP prime-boost strategy as a public health intervention against a possible H5N1 pandemic.

## Introduction

In the past century, three influenza pandemics have caused significant human fatalities throughout the world. Since 1997, highly pathogenic avian influenza (HPAI) H5N1 viruses have been spreading to numerous countries in Asia, Europe and Africa and infecting a large number of poultry and an increasing number of humans, often with lethal effects [1]–[3]. As of November 19, 2010, 508 human H5N1 infections have been confirmed, resulting in 302 deaths [4]. Although so far HPAI H5N1 transmission was found mostly via avian to human, continuous adaptation and/or re-assortment of HPAI H5N1 viruses may result in new strains capable of efficient human to human transmission. As a result, these viruses could cause significant morbidity and mortality, since humans are immunologically naïve to HPAI H5N1 viruses.

On the basis of hemagglutinin (HA) sequences, 10 clades of H5N1 viruses have emerged in various host species since 2000 [5]. Among them, clade 2 is divided into 5 subclades and subclade 2.3 is further divided into 2.3.1, 2.3.2, 2.3.3 and 2.3.4 [5]. So far the circulating HPAI H5N1 viruses of human isolates fall into clades 0, 1, 2 and 7 and the most recent human isolates in China belong to subclade 2.3.4 [6]. Increasingly, subclade 2.3.4 is also becoming one of dominant strains in poultry and birds in Southeast and East Asia [7].

The development of vaccine against HPAI H5N1 viruses has been impeded by its apparent poor immunogenicity [8]–[12]. In addition, the bio-safety concerns arise for the large-scale production of viruses required for conventional inactivated and live attenuated vaccines that would have potential risks of genetic exchange with circulating influenza virus strains [13]–[14]. To overcome these difficulties and concerns, currently many other vaccine strategies against HPAI H5N1 viruses are being developed in various stages [15], including replication incompetent human adenoviral vector [16]–[17], recombinant fowlpox viruses [18], recombinant new castle disease viruses [19], virus like particles (VLP) [20]–[25], retroviral pseudotypes [26], DNA plasmids [27]–[33] and recombinant proteins [34]–[35].

Although DNA plasmid and VLP vaccines have been individually tested against HPAI H5N1 viruses, as far as we are aware the combination of both vaccines into a heterologous prime-boost strategy against HPAI H5N1 viruses has not been reported before. Therefore, in this study we generated DNA plasmids expressing H5HA derived from a human H5N1 isolate (A/Shenzhen/406H/06, subclade 2.3.4) and VLP expressing H5HA and N1NA from the same isolate and compared neutralization titers and immune protection against lethal challenge of homologous and heterologous H5N1 viruses elicited with heterologous DNA-VLP versus homologous DNA-DNA and VLP-VLP prime-boost strategies in mice. Here we report that superior neutralizing antibody response and clinical efficacy were found in mice with heterologous DNA-VLP prime-boost strategy against high lethal dose challenge of HPAI H5N1 viruses.

## Methods

### Animals

All animal protocols were approved by the Institutional Animal Care and Use Committee at the Institut Pasteur in Cambodia. Female BALB/c mice (Mus musculus) at the age of 6 to 8 weeks were purchased from Charles River Laboratories (L'Arbresle, France) and housed in micro-isolator cages ventilated under negative pressure with HEPA-filtered air and a 12/12-hour light/dark cycle. Virus challenge studies were conducted in bio-safety level 3 (BSL-3) facility at the Institut Pasteur in Cambodia. Before each inoculation or euthanasia procedure, the mice were anesthetized by intraperitoneal injection of pentobarbital sodium (75 mg/kg, Sigma).

### Cell lines

The packaging cell line 293T was maintained in complete DMEM medium [i.e. high glucose DMEM supplemented with 10% FBS, 2 mM L-glutamine, 1 mM sodium pyruvate, penicillin (100 U/ml),) and streptomycin (100 µg/ml); Invitrogen Life Technologies] containing 0.5 mg/ml of G418. Madin-Darby canine kidney (MDCK) cell line was maintained in complete DMEM medium.

### Viruses

HPAI H5N1 viruses A/Shenzhen/406H/06 and A/Cambodia/P0322095/05 were originally isolated from human patients at the Donghu Hospital in Shenzhen, China and at the Institut Pasteur in Cambodia, respectively [36]–[37]. Viruses were propagated in MDCK cells and virus-containing supernatants were pooled, clarified by centrifugation and stored in aliquot at −80°C. The 50% tissue culture infection dose (TCID_50_) was determined by serial titration of viruses in MDCK cells and was calculated by the method of Reed and Muench [38]. To determine 50% mouse lethal dose (MLD_50_) of the viruses, groups of 5 mice were inoculated intranasally (i.n.) with serial 10-fold dilution of virus. After the inoculation, mice were monitored daily for clinical signs for 14 days. Mice that lost more than 25% of their original body weight were euthanatized and counted as dead. MLD_50_ was calculated by the method of Reed and Muench [38]. All research with HPAI H5N1 viruses was conducted under BSL-3 laboratory containment.

### Generation of HA and NA pseudotypes

Transfer vector pHR'CMV-Luc [39], packaging vector pCMVRΔ8.2 [40] and vectors encoding codon optimized H5HAs (A/Cambodia/P0322095/05 and A/Shenzhen/406H/06) and flag-epitope-tagged N1NA (A/Thailand/1(KAN-1)/04) were described before [41]. HA and NA pseudotypes were generated as described before [41].

### HA and NA pseudotype-based neutralization assay

HA and NA pseudotype-based neutralization assay was described before [41]. Briefly, MDCK cells (2×10^4^ cells per well) were seeded onto 24 well culture plate in complete DMEM overnight. Serially 2-fold diluted serum samples (starting at 1∶20 dilution) were incubated with HA and NA pseudotypes at the final volume of 100 µl at 37°C for 1 hr. The amount of HA and NA pseudotypes added corresponded to 200,000 relative luciferase activity (RLA). The mixture was added onto MDCK cells. After overnight incubation, cells were washed with PBS and cultured in complete DMEM medium. RLA was measured in 48 hrs by a BrightGlo Luciferase assay according to the manufacturer's instruction (Promega). The percentage of inhibition was calculated by (RLA in pseudotypes and medium control – RLA in pseudotypes and immune serum in a given dilution)/RLA in pseudotypes and medium control. IC50, IC90 and IC95 were determined as the dilutions of a given immune serum that resulted in 50, 90 and 95% reduction of RLA.

### Production and characterization of VLP

To generate VLP, 4.5×10^6^ 293T cells were co-transfected with 14 µg pCMVΔR8.2, 2 µg CMV/R-H5HA (A/Shenzhen/406H/06) and 0.5 µg CMV/R-flag epitope-tagged N1NA (A/Thailand/1(KAN-1)/04) using a calcium phosphate precipitation method as described before [41]. After overnight incubation, cells were washed once with PBS and cultured in 10 ml of complete DMEM supplemented with 100 µM sodium butyrate for 8 hrs. Cells were then cultured in 10 ml of complete DMEM. The VLP-containing supernatants were harvested in 16 to 20 hrs, loaded onto 20% sucrose cushion and ultra-centrifuged at 20,000 rpm for 2.5 hours at 4°C in a Beckman SW28 rotor (Beckman Coulter, Fullerton, CA). The pellets were resuspended in PBS and stored at −80°C in aliquots. To characterize VLP, resuspended pellets were further fractionated through a 25–65% sucrose density gradient at 25,000 rpm for 16 hours at 4°C in a Beckman SW41 rotor (Beckman Coulter, Fullerton, CA). Eleven fractions (1 ml each) were collected from the top to the bottom of the gradient, TCA precipitated, separated by 12% SDS-PAGE and transferred onto PDVF membranes. Blots were blocked in a solution of Tris-buffered saline containing 5% nonfat dry milk and 0.05% Tween 20 and subsequently probed with a monoclonal antibody (clone 183-H12) specific for HIV-1 gag p24 (the AIDS Reagents and Depositary program, NIAID, NIH), with a monoclonal antibody specific for flag epitope (Sigma) and with immune sera specific against H5HA [41]. Antigens were visualized with an AP-conjugated anti-mouse IgG antibody according to manufacturer's instruction (Promega).

### Electron microscopy

Electron microscopy to visualize VLP was done as described before [41].

### Immunization and challenge

Three sets of immunization and challenge experiments were carried out. In the first experiment, female BALB/c mice were randomly divided into 4 groups. Group one was injected intramuscularly (i.m.) of both hind legs with total 200 uL PBS (pH 7.4) in both prime and boost. Group two was injected i.m. with 0.4 ug (based on HA content) HA and NA VLP in total 200 ul PBS for both prime and boost. Group three was injected i.m. with 100 ug of plasmid DNA encoding H5 HA for both prime and boost. And the last group was primed i.m. with 100 ug of plasmid DNA encoding H5 HA and boosted i.m. with 0.4 ug HA and NA VLP. The prime was done on day 0 and the boost on day 21. Seven days before the prime and 7 days after the boost serum samples were collected, heat-inactivated at 56°C and stored in aliquots at −80°C. Two weeks after the boost, mice in each group were challenged i.n. with 10 MLD_50_ of homologous H5N1 virus (A/Shenzhen/406H/06, subclade 2.3.4) and heterologous H5N1 virus (A/Cambodia/P0322095/05, clade 1) in a volume of 50 ul. Mice were monitored and recorded daily for signs of illness, such as lethargy, ruffled hair and weight loss. Four days post challenge one mouse from each immunization and challenge group were sacrificed and lung tissues were taken for histopathologic evaluation (see below). For the remaining mice, when they lose 30% or more of their original weight, they were euthanized and counted as dead.

Since the results of the first experiment showed good protection against 10 MLD_50_ of homologous and heterologous H5N1 challenge in all three vaccine strategies, we carried out the second immunization and challenge experiment, in which all the procedures are the same as the first experiment except for 1,000 MLD_50_ of homologous and heterologous H5N1 were used for challenge.

To test whether DNA priming induces memory cell responses, we carried out the third immunization and challenge experiment, in which female BALB/c mice were randomly divided into 2 groups. Group one was injected with PBS. Group two was primed with plasmid DNA and boosted with VLP as described above. Sixty days after the boost, mice were challenged with 1,000 MLD_50_ of homologous H5N1 virus and monitored and recorded as described above.

All procedures were in accordance with the Department of Agriculture guidelines for the Care and Use of Laboratory Animals, the Animal Welfare Act and Department of Agriculture Bio-safety Guidelines in Microbiological and Biomedical Laboratory.

### Histopathologic evaluation

Lung tissues from infected mice were fixed in 4% neutral-buffered, ice cold paraformaldehyde, routinely processed and embedded in paraffin. Tissue sections were stained with hematoxylin and eosin (HE) for lesion detection.

### Statistical Analysis

Each individual animal immune response was counted as an individual value for statistical analysis. The significance of the immune response was calculated by Student's *t* test (tails  = 2 and type  = 2).

## Results

### Characterization of Immunogens

**Figure 1:**
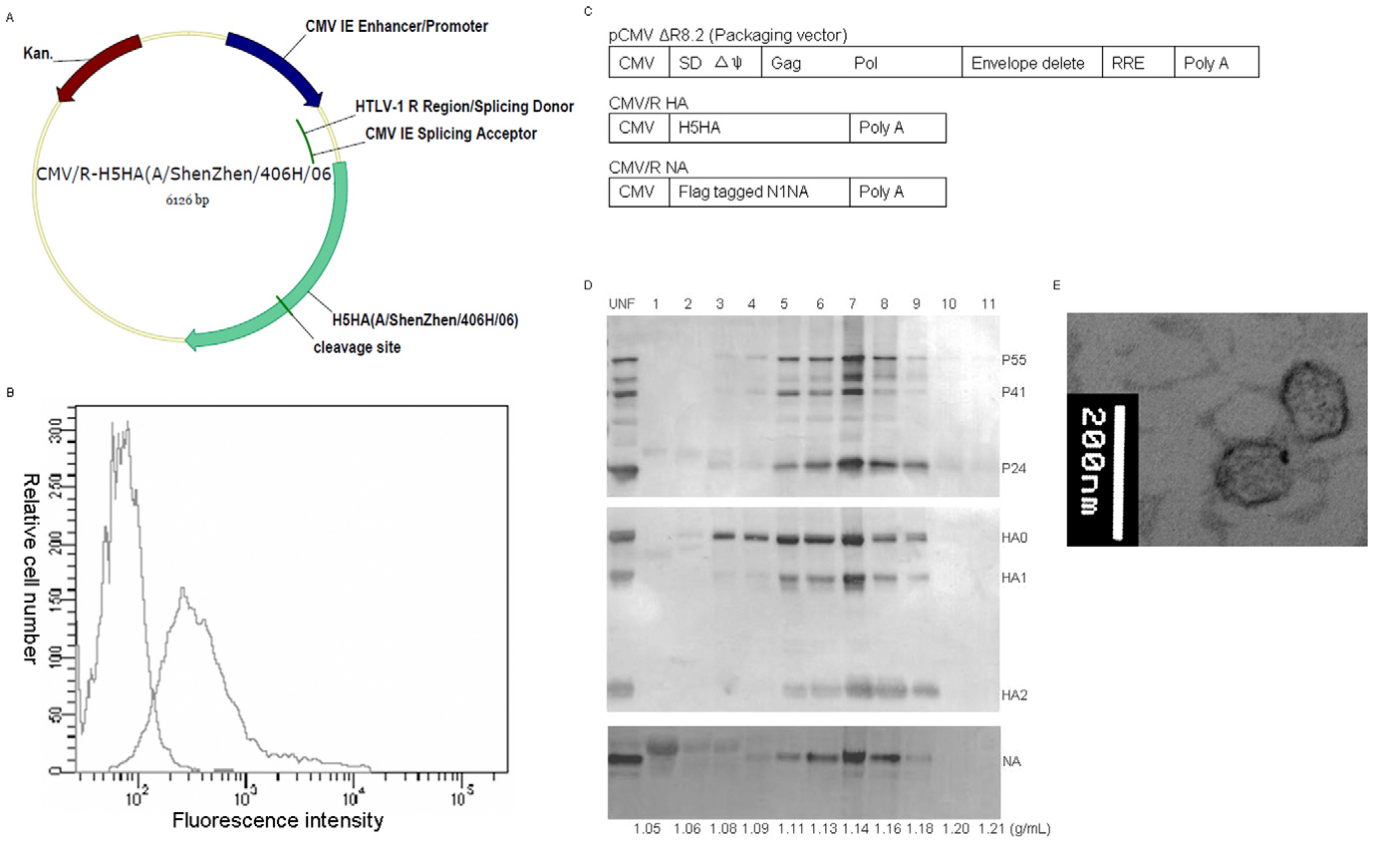
Characterization of immunogens. A) Schematic diagram of DNA plasmid vector expressing H5HA (clade 2.3.4). B) Cell surface expression of H5HA protein on 293 T cells transiently transfected with the DNA plasmid vector expressing H5HA (A/Shenzhen/406H/06, clade 2.3.4) detected by anti-H5HA-specific immune sera. C) Schematic diagram of DNA plasmids expressing H5HA (A/Shenzhen/406H/06, clade 2.3.4), flag epitope-tagged N1NA and HIV-1 gag/pol used for making influenza H5HA and N1NA VLP. D) Western blot analysis of influenza HA, NA and HIV-1 gag proteins in 12 fractions after sucrose gradient fractionation of influenza H5HA and N1NA VLP detected by anti-H5HA-specific immune sera, anti-flag epitope antibody and anti-HIV-1 gag p24 antibody. E) Influenza H5HA and N1NA VLPs revealed by electron microscopy.

Figure 1A shows the schematic diagram of DNA plasmid expression vector encoding codon optimized H5HA (A/Shenzhen/406H/06, subclade 2.3.4). To determine the expression of H5HA, 293T cells were transiently transfected with DNA plasmids followed by immune staining of anti-H5HA immune sera. Figure 1B shows that H5HA is expressed on the surface of transfected 293T cells.

Figure 1C shows the schematic diagram of three expression vectors encoding H5HA, N1NA and HIV-1 gag/pol used to generate VLP (see Methods for the detail). VLP containing supernatants were harvested, concentrated and fractionated by sucrose density gradient centrifugation. The incorporation of H5HA and N1NA into VLP was analyzed by Western blot. Figure 1D shows the H5HA, and N1NA proteins along with HIV-1 gag were detected in the same fractions at buoyant density between 1.09 and 1.18. The co-migration of H5HA and N1NA with HIV-1 gag in the sucrose density gradient indicates that both H5HA and N1NA are incorporated into VLP. Figure 1E shows an electronmicrograph of H5N1 and N1NA VLP.

### Clinical outcome of mice after the immunization and challenge

**Figure 2:**
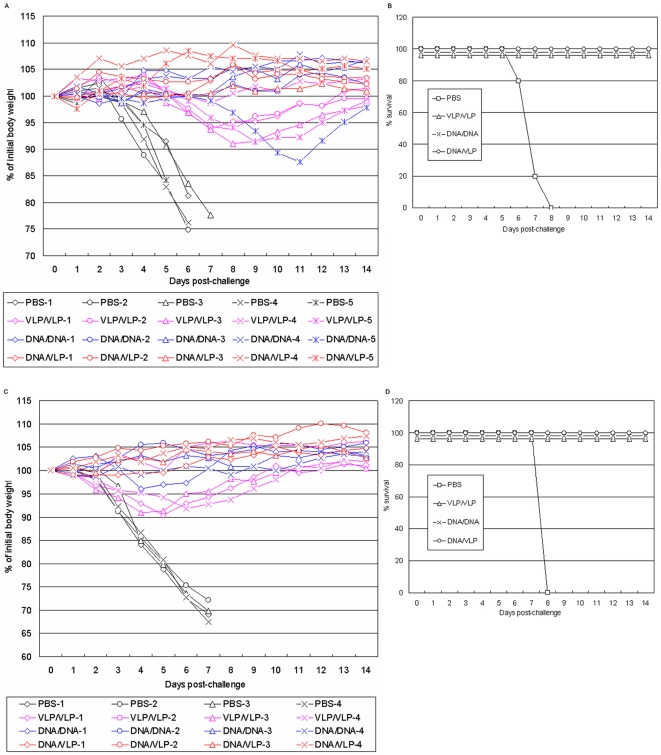
Comparison of vaccine efficacy elicited with heterologous DNA-VLP prime-boost versus homologous DNA-DNA, VLP-VLP prime-boost strategies in mice following 10 MLD_50_ of homologous (A/Shenzhen/406H/06, clade 2.3.4) and heterologous (A/Cambodia/P0322095/05, clade 1) H5N1 virus challenge. A) Percentage of original body weight in individual mice after challenged with 10 MLD_50_ of homologous (A/Shenzhen/406H/06, clade 2.3.4) H5N1 virus. Black color: PBS control group; red color: heterologous DNA-VLP prime-boost group; pink color: homologous VLP-VLP prime-boost group; blue color: homologous DNA-DNA prime-boost group. B) Survival rates after challenged with 10 MLD_50_ of homologous (A/Shenzhen/406H/06, clade 2.3.4) H5N1 virus. Survival rate was calculated based on percent survival within each experimental group (n = 5 mice per experimental group; *, P<0.05, Kaplan-Meier survival analysis). Open square: PBS control group; open triangle: homologous VLP-VLP prime-boost group; open circle: heterologous DNA-VLP prime-boost group; cross: homologous DNA-DNA prime-boost group. C) Percentage of original body weight in individual mice after challenged with 10 MLD_50_ of heterologous (A/Cambodia/P0322095/05, clade 1) H5N1 virus. Black color: PBS control group; red color: heterologous DNA-VLP prime-boost group; pink color: homologous VLP-VLP prime-boost group; blue color: homologous DNA-DNA prime-boost group. D) Survival rates after challenged with 10 MLD_50_ of heterologous (A/Cambodia/P0322095/05, clade 1) H5N1 virus. Survival rate was calculated based on percent survival within each experimental group (n = 4 mice per experimental group).

To compare protective effect of heterologous versus homologous prime-boost strategies against H5N1 virus challenge, in the first experiment, the VLP-VLP, DNA-DNA and DNA-VLP vaccinated mice along with PBS control mice were challenged i.n. with 10 MLD_50_ of homologous H5N1 virus (A/Shenzhen/406H/06, subclade 2.3.4) and heterologous H5N1 virus (A/Cambodia/P0322095/05, clade 1) (Table 1). Dose 10 MLD_50_ was chosen to ensure 100% mortality rates in PBS control. Figure 2 show the time course of body weight change in individual mice and the survival rate in the four groups of mice after challenged with 10 MLD_50_ of homologous and heterologous H5N1 viruses.

**Table 1:** Immunization and challenge schedule.

Time	Group			
	PBS	DNA-DNA	DNA-VLP	VLP-VLP
Day -7	Bleeding	Bleeding	Bleeding	Bleeding
Day 0	Immunized with PBS	Immunized with DNA	Immunized with DNA	Immunized with VLP
Day 21	Immunized with PBS	Immunized with DNA	Immunized with VLP	Immunized with VLP
Day 28	Bleeding	Bleeding	Bleeding	Bleeding
Day 35	Infected with 10 MLD_50_ H5N1 A/Shenzhen/406H/06 and A/Cambodia/P0322095/05 or 1000 MLD_50_ H5N1 A/Shenzhen/406H/06 and A/Cambodia/P0322095/05			
Day 39	Sacrifice one mouse of each group challenged with 10 and 1000 MLD_50_ H5N1A/Shenzhen/406H/06 virus and the lungs were removed and fixed			
Day 35–48	Weight and survival were monitored and recorded daily			

After challenged with 10 MLD_50_ of homologous HPAI H5N1 virus, mice in PBS group became sick, as evidenced by rough coat, less reactive, passive during handling, rolled up and labored breath, on day 3 after the challenge, rapidly lost weight. All mice died between day 6 and 8. Mice in VLP-VLP group became sick on day 4, lost weight between day 6 and 8. However, starting on day 10 they regained weight and all mice survived. In DNA-DNA group, only one mouse (#5) significantly lost weight and all mice survived. In contrast, in DNA-VLP group after challenge, no mice had any sign of illness and weight loss and all survived (Figure 2A and 2B). After challenged with 10 MLD_50_ of heterologous HPAI H5N1 virus, similar pattern of illness, weight loss and survival were observed among the four groups of mice (Figure 2C and 2D).

**Figure 3:**
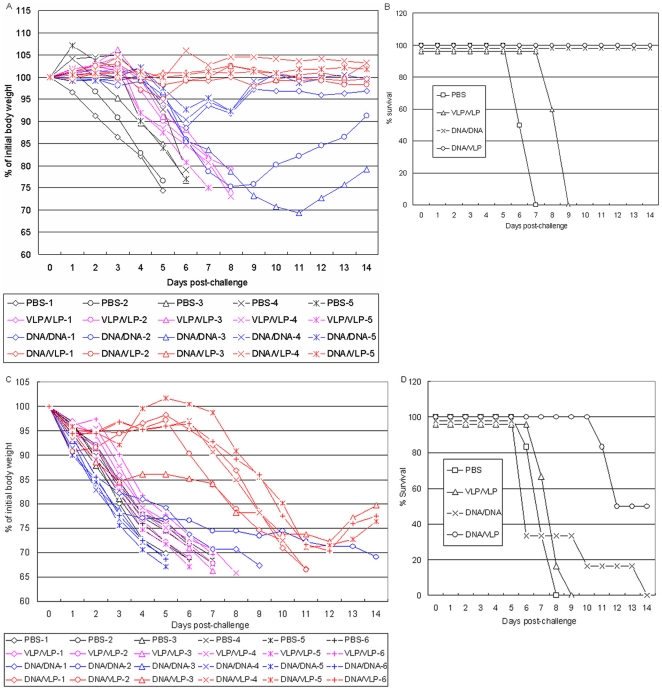
Comparison of vaccine efficacy elicited with heterologous DNA-VLP prime-boost versus homologous DNA-DNA, VLP-VLP prime-boost strategies in mice following 1000 MLD_50_ of homologous (A/Shenzhen/406H/06, clade 2.3.4) and heterologous (A/Cambodia/P0322095/05, clade 1) H5N1 virus challenge. A) Percentage of original body weight in individual mice after the 1,000 MLD_50_ of homologous H5N1 virus challenge. Black color: PBS control group; red color: heterologous DNA-VLP prime-boost group; pink color: homologous VLP-VLP prime-boost group; blue color: homologous DNA-DNA prime-boost group. B) Survival rates after the 1,000 MLD_50_ of homologous H5N1 virus challenge. Survival rate was calculated based on percent survival within each experimental group (n = 5 mice per experimental group). Open square: PBS control group; open triangle: homologous VLP-VLP prime-boost group; open circle: heterologous DNA-VLP prime-boost group; cross: homologous DNA-DNA prime-boost group. C) Percentage of original body weight in individual mice after challenged with 1000 MLD_50_ of heterologous (A/Cambodia/P0322095/05, clade 1) H5N1 virus. Black color: PBS control group; red color: heterologous DNA-VLP prime-boost group; pink color: homologous VLP-VLP prime-boost group; blue color: homologous DNA-DNA prime-boost group. D) Survival rates after challenged with 1000 MLD_50_ of heterologous (A/Cambodia/P0322095/05, clade 1) H5N1 virus. Survival rate was calculated based on percent survival within each experimental group (n = 6 mice per experimental group).

Since no signs of illness were observed in DNA-VLP group after challenged with 10 MLD_50_ homologous and heterologous HPAI H5N1 viruses, in the second experiment, mice were challenged with 1,000 MLD_50_ of homologous and heterologous HPAI H5N1 virus. Figure 3A shows the time course of body weight change in individual mice and Figure 3B shows the survival rate of the four groups of mice after the homologous challenge. In PBS group, mice became sick on day 1 after the challenge and rapidly lost weight. Between day 6 and 7 all mice died. In VLP-VLP group, mice became sick on day 3, lost weight between day 4 and 8, and died on day 8 and 9. In DNA-DNA group, mice became sick on day 4, lost weight between day 5 and 11. Interestingly, three mice (#1, #4 and #5) in this group then regained weight on day 9, another one (#2) on day 11 and still another one (#3) on day 12. All mice survived. In contrast, in DNA-VLP group, again no mice show any visible signs of illness and weight loss despite such a high lethal dose challenge.

Figure 3C shows the time course of body weight change in individual mice and Figure 3D shows the survival rate of the four groups of mice after 1,000 MLD50 of heterologous H5N1 challenge. In PBS and VLP-VLP groups, mice became sick on day 1 after the challenge and rapidly lost weight. Between day 5 and 7 all mice died. Between day 5 and 13 all mice died in DNA-DNA group. In contrast, in DNA-VLP group, except for one mouse (#3) all mice lost weight much later and 3 of 6 mice actually survived.

**Figure 4:**
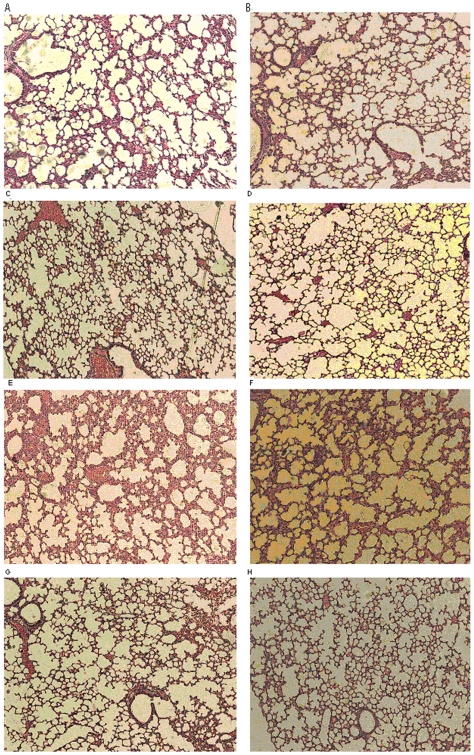
Histological lesions of lung tissue detected by hematoxylin and eosin (HE) staining in mice 4 days post-challenge with 10 MLD_50_ (A to D) or 1,000 MLD_50_ (E to H) of homologous (A/Shenzhen/406H/06, clade 2.3.4) H5N1 virus. A and E) PBS control group; B and F) homologous VLP-VLP prime-boost group; C and G) homologous DNA-DNA prime-boost group; D and H) heterologous DNA-VLP prime-boost group.

To better understand the effect of homologous versus heterologous prime-boost strategies on clinical outcome of HPAI H5N1 virus challenge, 4 days after the challenge of 10 and 1,000 MLD_50_ of homologous HPAI H5N1 virus, one mouse in each group was sacrificed and lung tissues were collected for pathologic evaluation. Figure 4 shows the HE-stained tissue sections of lung taken on day 4 post challenge with 10 and 1,000 MLD_50_ of homologous HPAI H5N1 virus. After 10 MLD_50_ challenge, lung tissue in PBS control mice exhibited interstitial pneumonia with hypertrophy of alveolar lining cells and lymphocyte infiltration as well as enlarged blood vessel (Figure 4A). Lung tissue in VLP-VLP immunization mice also exhibited interstitial pneumonia with less severity (Figure 4B). In contrast, lung tissues in DNA-DNA and DNA-VLP immunization mice exhibited spongiform aspect of the lungs with well-delineated, thin alveolar septa and without any sign of interstitial pneumonia (Figure 4C and 4D).

After 1,000 MLD_50_ challenge, lung tissue in PBS control and VLP-VLP immunization mice both exhibited severe interstitial pneumonia with alveolar septa thickened by high degree of lymphocyte infiltration as well as enlarged blood vessel full of red blood cells (Figure 4E and 4F). Lung tissue in DNA-DNA immunization mice also exhibited interstitial pneumonia with much less severity (Figure 4G). In contrast, lung tissues in DNA-VLP immunization mice had no sign of interstitial pneumonia (Figure 4H). Taken together, both clinical and histopathological results strongly suggest that heterologous DNA-VLP prime-boost immunization completely protects mice from infection caused by 10 MLD_50_ heterologous and 1,000 MLD_50_ of homologous H5N1 challenge and partially protects mice from death caused by 1,000 MLD_50_ of heterologous H5N1 challenge.

**Figure 5:**
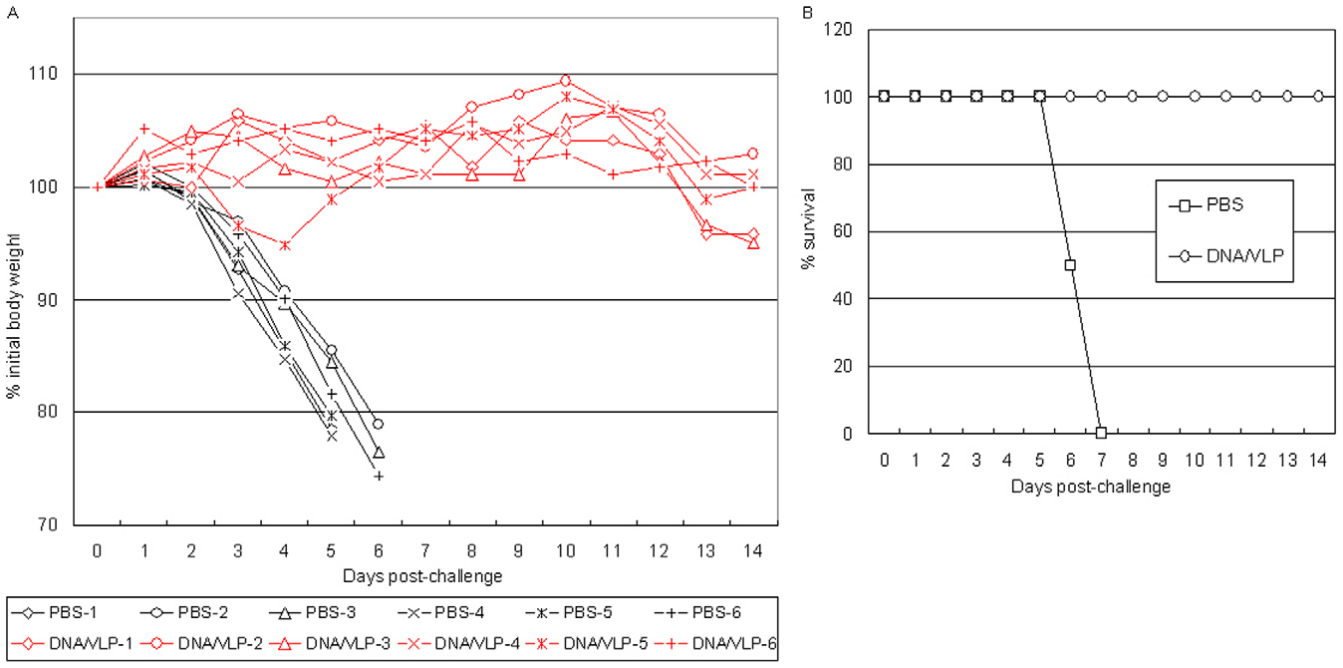
Vaccine efficacy elicited with heterologous DNA-VLP prime-boost strategy in mice following 1000 MLD_50_ of homologous (A/Shenzhen/406H/06, clade 2.3.4) at 60 days post boost. A) Percentage of original body weight in individual mice after the 1,000 MLD_50_ of homologous H5N1 virus challenge. Black color: PBS control group; red color: heterologous DNA-VLP prime-boost group. B) Survival rates after the 1,000 MLD_50_ of homologous H5N1 virus challenge. Survival rate was calculated based on percent survival within each experimental group (n = 6 mice per experimental group).

To test potential memory response elicited by DNA-VLP immunization, we carried out the third immunization and challenge experiment, in which one group of female BALB/c mice was injected with PBS and the other group was primed with plasmid DNA and boosted with VLP as described above. We then rested mice for sixty days before challenging them with 1,000 MLD_50_ of homologous H5N1 virus. As shown in Figure 5, no significant weight loss (Figure 5A) and no death were observed in DNA-VLP group (Figure 5B).

### Neutralizing antibody responses against homologous and heterologous H5N1 viruses

To gain insight into the mechanism of protection, we compared neutralizing antibody titers in pre-prime, post-boost and post-challenge sera using a HA and NA pseudotype-based neutralization assay. Table 2 and 3 summarize that IC50, IC90 and IC95 of individual serum samples from these four groups of mice against homologous (A/Shenzhen/406H/06, subclade 2.3.4) and heterlogous (A/Cambodia/P0322095/05, clade 1) H5N1 pseudotypes, respectively.

**Table 2:** Neutralizing antibody titers against homologous H5N1 pseudotype virus A/Shenzhen/406H/06.

		Pre-immune sera		Post-immune sera		Post-challenge sera	
	NO	IC50	IC95	IC50	IC95	IC50	IC95
PBS	1	<1:10*	<1:10	<1:10	<1:10	ND**	ND
	2	<1:10	<1:10	<1:10	<1:10	ND	ND
	3	<1:10	<1:10	<1:10	<1:10	ND	ND
	4	<1:10	<1:10	<1:10	<1:10	ND	ND
	5	<1:10	<1:10	<1:10	<1:10	ND	ND
	6	<1:10	<1:10	<1:10	<1:10	ND	ND
VLP/VLP	1	<1:10	<1:10	746	<1:10	943	50
	2	<1:10	<1:10	43	<1:10	990	10
	3	<1:10	<1:10	10	<1:10	943	49
	4	<1:10	<1:10	153	<1:10	980	125
	5	<1:10	<1:10	1493	<1:10	1087	53
	6	<1:10	<1:10	296	<1:10	ND	ND
DNA/DNA	1	<1:10	<1:10	1754	22	2632	159
	2	<1:10	<1:10	1724	50	926	159
	3	<1:10	<1:10	2128	10	1010	98
	4	<1:10	<1:10	2564	24	714	54
	5	<1:10	<1:10	472	<1:10	1124	96
	6	<1:10	<1:10	1333	10	ND	ND
DNA/VLP	1	<1:10	<1:10	4167	56	>10240	281
	2	<1:10	<1:10	2941	49	4545	141
	3	<1:10	<1:10	3333	51	4545	63
	4	<1:10	<1:10	2641	63	4545	145
	5	<1:10	<1:10	2564	56	>10240	198
	6	<1:10	<1:10	8333	186	ND	ND

*<1:10: Not detectable.

**ND: Not done.

**Table 3:** Neutralizing antibody titers against heterologous H5N1 pseudotype virus A/Cambodia/P0322095/05.

		Pre-immune sera		Post-immune sera		Post-challenge sera		
	NO	IC50	IC95	IC50	IC95	IC50	IC90	IC95
PBS	1	<1:10*	<1:10	<1:10	<1:10	ND**	ND	ND
	2	<1:10	<1:10	<1:10	<1:10	<1:10	<1:10	<1:10
	3	<1:10	<1:10	<1:10	<1:10	ND	ND	ND
	4	<1:10	<1:10	<1:10	<1:10	ND	ND	ND
VLP/VLP	1	<1:10	<1:10	<1:10	<1:10	78	<1:10	<1:10
	2	<1:10	<1:10	<1:10	<1:10	212	10	<1:10
	3	<1:10	<1:10	<1:10	<1:10	60	<1:10	<1:10
	4	<1:10	<1:10	<1:10	<1:10	136	<1:10	<1:10
DNA/DNA	1	<1:10	<1:10	<1:10	<1:10	346	18	<1:10
	2	<1:10	<1:10	<1:10	<1:10	190	14	<1:10
	3	<1:10	<1:10	<1:10	<1:10	402	10	<1:10
	4	<1:10	<1:10	<1:10	<1:10	139	<1:10	<1:10
DNA/VLP	1	<1:10	<1:10	<1:10	<1:10	1299	86	22
	2	<1:10	<1:10	<1:10	<1:10	617	38	<1:10
	3	<1:10	<1:10	<1:10	<1:10	556	25	<1:10
	4	<1:10	<1:10	<1:10	<1:10	306	10	<1:10

*<1:10: Not detectable.

**ND: Not done.

As expected, pre-prime sera and sera from PBS control mice had no detectable neutralizing antibody titers against both pseudotypes. Post-boost sera elicited with VLP-VLP showed moderate neutralizing antibody titers against homologous H5N1 pseudotypes with IC50 ranging from 1∶10 to 1∶1,493. Post-boost sera elicited with DNA-DNA showed higher neutralizing antibody titers against homologous pseudotypes with IC50 ranging from 1∶472 to 1∶2,564 and with IC95 in four mice ranging from 1∶10 to 1∶50. Post-boost sera elicited with DNA-VLP exhibited the highest neutralizing antibody titers against homologous pseudotypes with IC50 ranging from 1∶2,564 to 1∶8,333 and IC95 ranging from 1∶49 to 1∶186 (Table 2). Interestingly, none of these post-boost sera exhibited neutralizing antibody activity against heterologous H5N1 pseudotypes (Table 3). Statistically, no matter whether it was calculated by IC50 or by IC95, the neutralizing antibody titers in post-boost sera between any of two groups were always significant. For example, when calculated by IC95, *P* value was 0.047 between VLP-VLP and DNA-DNA; 0.008 between VLP-VLP and DNA-VLP; and 0.033 between DNA-DNA and DNA-VLP.

After the challenge with homologous virus, neutralizing antibody titers were significantly increased with IC95 ranging from 1∶10 to 1∶125 in VLP-VLP group; from 1∶54 to 1∶159 in DNA-DNA group; and from 1∶63 to 1∶281 in DNA-VLP group (Table 2). Only *P* value that is statistically significant is between VLP-VLP and DNA-VLP (*P* = 0.028). Importantly, after the challenge with heterologous virus, neutralizing antibody titers against heterologous pseudotypes became detectable in all three prime-boost groups with IC50 ranging from 1∶60 to 1∶212 in VLP-VLP group; from 1∶190 to 1∶402 in DNA-DNA group; and from 1∶306 to 1∶1,299 in DNA-VLP group (Table 3). Only *P* value that is statistically significant is also between VLP-VLP and DNA-VLP (*P* = 0.037).

## Discussion

The emergence of H5N1 influenza virus to which the human population has little immunity raises great public health concern. Although vaccination is the most economically prudent public health intervention strategy against both seasonal and pandemic influenza, to date, clinical evaluation of H5N1 vaccine candidates indicate the need for alternative approaches that could enhance vaccine immunogenicity and better protection. In the present study, we compared neutralizing antibody responses and immune protection elicited with heterologous DNA-VLP, homologous DNA-DNA and VLP-VLP prime-boost strategies against HPAI H5N1 viruses in mice. We demonstrate that DNA-VLP elicits the highest neutralizing antibody titers; whereas DNA-DNA elicits higher neutralizing antibody titers than VLP-VLP (Table 2 and Table 3). We show that although all three prime-boost strategies protect mice from death caused by 10 MLD_50_ of homologous and heterologous H5N1 challenge, only DNA-VLP and DNA-DNA protect mice from infection as manifested by no weight loss and no lung pathology (Figures 2 and 4); and although DNA-VLP and DNA-DNA protect mice from death caused by 1,000 MLD_50_ of homologous H5N1 challenge, only DNA-VLP protects mice from infection (Figures 3A and 3B and 4). Moreover, we show that after 1,000 MLD_50_ of heterologous H5N1 challenge, while all mice in PBS, VLP-VLP and DNA-DNA died, 3 of 6 mice in DNA-VLP actually survived (Figure 3C and 3D). Finally, we show that DNA-VLP completely protects mice from infection after 1,000 MLD_50_ of homologous H5N1 challenge even when the challenge was administrated at 60 days post the boost (Figure 5). Thus, we conclude that heterologous DNA-VLP prime-boost strategy is superior to homologous DNA-DNA and VLP-VLP strategies against HPAI H5N1 viruses.

Although the present study is the first report on heterologous DNA-VLP prime-boost strategy against H5N1 viruses, heterologous prime-boost strategies with other combinations of different antigen-delivery systems have been extensively used against H5N1 viruses as well as seasonal and pandemic influenza viruses [42]–[48]. For example, Ramshaw *et al.* showed that mice primed with DNA encoding HA and boosted with a recombinant fowlpox virus produced extremely high levels of anti-HA serum antibodies, predominantly of the IgG2a isotype, and were protected against homologous influenza virus challenge [42]. Ikeno *et al.* tested prime-boost strategies against H5N1 viruses by priming mice with an adjuvanted inactivated whole clade 1 H5N1 vaccine and boosting with split or whole inactivated clade 2 H5N1 vaccine with or without adjuvant [43]. Lo *et al.* compared immunization with cold-adapted viruses to DNA prime and recombinant adenovirus boost immunization for the induction of heterosubtypic immune protection against H5N1 virus [44]. Wang *et al.* showed that heterologous DNA prime and inactivated vaccine boost immunization was more effective than using DNA or inactivated vaccine alone against seasonal influenza viruses [45]. Steensels *et al.* showed that priming Perkin ducks with a fowlpox vector and then boosting with an inactivated avian influenza vaccine resulted in higher immunogenicity and full protection against HPAI H5N1 virus [46]. Pan *et al.* tested DNA prime and inactivated vaccine boost in chicken and found the vaccination strategy completely protected animals from HPAI H5N1 challenge [47]. Thus, our present study and studies by other investigators clearly demonstrate that heterologous prime-boost strategies are very effective ways to induce high levels of immune responses and better protection.

While the present study was not designed to conduct an in-depth immunological analysis on the mechanism(s) of why heterologous DNA-VLP prime-boost strategy would be more effective than homologous DNA-DNA and VLP-VLP prime-boost strategies, our results clearly show that heterologous DNA-VLP prime-boost strategy does elicit much higher neutralizing antibody titers than homologous DNA-DNA and VLP-VLP prime-boost strategies do; and the levels of neutralization titers in post-boost and/or post-challenge sera correlate with better clinical outcome. Thus, a better understanding why heterologous DNA-VLP prime-boost strategy elicits higher neutralizing antibody responses is crucial for the development of more effective vaccination strategies. It is tempting to speculate that DNA prime, due to its low, but longer lasting, antigen delivery, could be more effective in eliciting antigen-specific memory B cells than VLP prime; while VLP boost, due to its particulate form, right conformation of HA and NA on the particle surface and non-replication nature, could be more effectively processed and presented by antigen presenting cells to induce appropriate CD4 T helper cells and to recall memory B cell response. Several studies on B cell immunology have shown that low dose antigen delivery effectively elicits better memory B cells [49]–[50]. Experiments are currently under way to determine whether this is indeed the case.
